# The Use of Rubber Dam

**Published:** 1891-08

**Authors:** Louis Ottofy

**Affiliations:** Chicago, Ill.


### The Use of Rubber Dam.

" I trust that no one is in the habit of using the same piece of rubber dam on different patients, and hence, I only refer *en passant* to the possibility of inoculation and the transmission of disease from one patient to another. This is notoriously probable when the rubber dam has been used in a mouth affected with pyorrhœa alveolaris. Indeed, it is possible to transfer the disease from one part of the mouth to another in the same person; hence, it should not be used a second time in the same mouth. Of course, I understand that the rubber dam is always washed before a second using, yet we know that disease germs are subtle and some of them even microscopically undistinguishable. Furthermore, it ought not to be used a second time in the same mouth because of the unpleasant sight of using a thing like that which does not look fresh; the dam is at best unsightly, and taking it from a book of blotters, or from an envelope with the pateint's name on the same, or from a row hanging on the wall with the patient's name stamped on each piece of rubber, or picking it out of the waste basket, memory and identification of the holes in the rubber acting as a guide for the identification of the rubber—neither of these methods is pleasant and should, therefore, not be resorted to, the cost of the rubber is almost nominal, and the expense should be considered as much a legitimate one as the gold introduced and also as an essential accompaniment of the filling, the cost each time being from 2½ to 5 cents. Considering an extreme case in which all possible operations may be performed in one I doubt that the expense of rubber dam could be more than $2.00, which would represent only a comparatively small portion of the entire expense."

LOUIS OTTOFY, D.D.S., Chicago, Ill.

